# A hidden threat: a case of relapsed disseminated *Mycobacterium abscessus* infection and its therapeutic challenges

**DOI:** 10.5194/jbji-11-185-2026

**Published:** 2026-03-30

**Authors:** Katherine Grace Egan, Lisa Duffy, Catherine Fleming, Padraig McGettrick, Eavan G. Muldoon, Christine Kelly, James Woo, Emer Kilbride, Joseph Butler, Richard Storey, Christine Quinlan, Edward McDermott, Jonathan Hunter, Carlos Mejia-Chew

**Affiliations:** 1 Department of Infectious Diseases, Mater Misericordiae University Hospital, Dublin, Ireland; 2 Department of Infectious Diseases, Galway University Hospital, Galway, Ireland; 3 University College Dublin School of Medicine, University College Dublin, Belfield, Dublin 4, Ireland; 4 Department of Orthopaedic Surgery, Mater Misericordiae University Hospital, Dublin, Ireland; 5 Department of Plastics & Reconstructive Surgery, Mater Misericordiae University Hospital, Dublin, Ireland; 6 Department of Radiology, Mater Misericordiae University Hospital, Dublin, Ireland

## Abstract

*Mycobacterium abscessus* (*M. abscessus*) is a rapidly growing nontuberculous mycobacterium (NTM). We present the case of a 57-year-old female on immunosuppressive therapy for polymyalgia rheumatica (PMR) who developed disseminated *M. abscessus* infection with vertebral osteomyelitis following bariatric surgery abroad. Her case highlights core treatment principles of disseminated NTM infections.

## 1 Introduction

*M. abscessus* is a rapidly growing nontuberculous mycobacterium (NTM) associated with opportunistic infections. Disseminated infection is rare and typically occurs in immunosuppressed hosts. The lipid-rich cell wall and ability to form biofilm enable persistence on medical devices, immune evasion, and intrinsic resistance to antimicrobials and disinfectants, making management exceptionally difficult (Lebeaux et al., 2014; Falkinham, 2020). There has been a global increase in NTM infections driven by multiple factors (Dahl et al., 2022; To et al., 2020). We present a case of an immunocompromised woman with disseminated *M. abscessus* infection with vertebral osteomyelitis and skin nodules following bariatric surgery abroad. The case highlights essential treatment principles in the management of these challenging infections.

## 2 Case

A 57-year-old woman with obesity and polymyalgia rheumatica (PMR) on alternate-day prednisolone (10–15 mg) presented with back pain and discharge from an abdominal drain site 1 week post-sleeve gastrectomy performed in Türkiye. She was prescribed oral amoxicillin–clavulanic acid by her general practitioner for a presumed surgical site infection; however, she then developed pyrexia, generalized abdominal pain, and worsening back pain, so she was brought to her local hospital.

An abdominal computed tomography (CT) scan demonstrated a 4.7×4.5×1.2 cm abdominal wall collection related to her laparoscopic port site, thought to be a seroma. Magnetic resonance imaging (MRI) of the thoracic and lumbar spine showed mild multilevel spondylitic changes without evidence of discitis. She was treated for a suspected pyelonephritis given reported dysuria, right flank tenderness on exam, and a urine culture with mixed organisms ( >100000 cfu per mL) and was discharged home.

**Figure 1:**
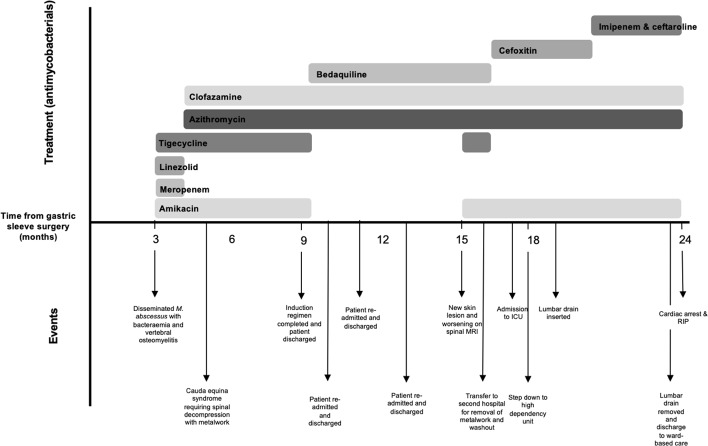
Illustration demonstrating antimycobacterial use over the patient's course along with main events.

**Figure 2:**
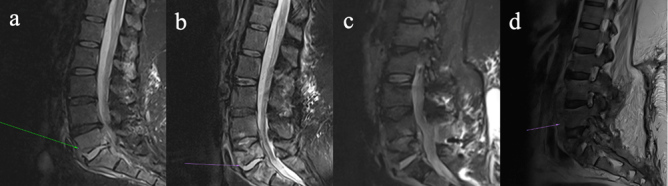
Four-panel figure demonstrating progressive discitis/osteomyelitis involving the lower lumbar spine on magnetic resonance imaging **(a–d)**. **(a)** Sagittal STIR images show discitis/osteomyelitis at L5/S1. **(b)** Sagittal STIR images now demonstrate progression of disease, with erosion of the L5 superior endplate, development of an anterior epidural collection, and associated ligamentum hypertrophy with resultant bunching of the distal cauda equina. **(c)** STIR image pre-instrumentation removal demonstrate a progressive discitis/osteomyelitis at L4/L5, with multiple enhancing collections within the anterior epidural space and posterior to the anterior longitudinal ligament. **(d)** Sagittal T2 images of the lumbar spine post-hardware removal demonstrate further destruction of the L5 vertebral body, with a persistent enhancing collection within the anterior epidural space, and a new collection along the anterior longitudinal ligament.

She re-presented 3 weeks later with pyrexia, drowsiness, and progressive lower back pain. A repeat MRI lumbar spine revealed L5/S1 osteomyelitis with bilateral multiloculated abscesses involving the iliopsoas muscles (Fig. 2a). Interventional radiology facilitated a biopsy of the L5/S1 disc space which identified *M. abscessus* complex. Peripheral blood cultures identified *Mycobacteria* species other than tuberculosis complex. Antimicrobial therapy (Fig. 1) was commenced with amikacin, linezolid, tigecycline, and meropenem, since imipenem and cefoxitin are not widely available in Ireland. Following multi-disciplinary discussion, surgical intervention was deferred as it was deemed high-risk given the absence of neurological symptoms. Based on drug susceptibility testing (DST) (Table 1), azithromycin and clofazimine were added to the regimen and linezolid was discontinued. Meropenem was stopped due to a suspected delayed hypersensitivity reaction. Following 3 weeks of antimicrobial treatment, repeat mycobacterial blood cultures were sterile.

**Table 1:** Samples that grew *Mycobacterium abscessus* and susceptibility testing over time.

Antimicrobial	Index	Skin	Surgical
	positive	biopsy	metalware
	lumbar	of left	removal
	bone	forearm	spinal
	biopsy	(348 d	tissue
	(day 0)	post-index)	(371 d
			post-index)
Clarithromycin	S	S	0.12
Amikacin	S	S	S
Linezolid	R	S	S
Tigecycline	0.5	0.12	0.25
Imipenem	I	R	I
Cefoxitin	n/a	n/a	I
Moxifloxacin	R	R	R
Ciprofloxacin	R	R	R
Co-trimoxazole	R	R	R
Doxycycline	R	R	R
Meropenem	n/a	32	n/a
Ceftaroline	n/a	64	n/a
Cefuroxime	n/a	32	n/a
Cefdinir	n/a	32	n/a
Imipenem + cefdinir	n/a	2	n/a
Imipenem + cefuroxime	n/a	1	n/a
Imipenem + ceftaroline	n/a	0.25	n/a
Meropenem + cefdinir	n/a	4	n/a
Meropenem + cefuroxime	n/a	0.25	n/a
Meropenem + Ceftaroline	n/a	0.5	n/a

Phenotypic drug susceptibility testing (broth microdilution) was performed by the Scottish mycobacteriology lab and reported as S = susceptible, I = intermediate, R = resistant. n/a = not applicable. Numbers represent the minimum inhibitory concentration in µ g mL^−1^.

Six weeks after starting antimicrobial treatment, she developed new loss of power in ankle dorsiflexion. A repeat MRI lumbar spine revealed an anterior epidural abscess at L4/L5, narrowing of the spinal canal, and bunching of the cauda equina (Fig. 2b). The acute cauda equina syndrome prompted emergent surgery for decompression and stabilization with pedicle screws and rods. Intra-operative tissue samples identified *Mycobacteria* species other than tuberculosis complex. Her dose of prednisolone was doubled to 30 mg prior to surgery as a stress dose which was tapered postoperatively. She completed induction antimicrobial regimen during her 4-month admission, and was discharged on azithromycin, clofazimine, and bedaquiline (Fig. 1).

She was readmitted to hospital on three further occasions for interventional radiology drainage of paraspinal abscesses seen in interval imaging. Many changes were made to her prednisolone dosing, and anakinra, an interleukin-1 receptor antagonist, was given as a steroid-sparing agent.

At Infectious Diseases clinic follow-up, she endorsed worsening back pain and new skin lesions. A biopsy of the skin lesions showed acid-fast bacilli (AFB) on stains and isolated *Mycobacterium abscessus* complex (Table 1). Repeat MRI lumbar spine (Fig. 2c) displayed an increase in the paraspinal subcutaneous fluid collection ( 5.0×2.2×13 cm), but repeat mycobacterial blood cultures showed no growth. Amikacin and tigecycline were restarted, and the patient was transferred to the National Spinal Unit for surgical evaluation. Upon transfer, she underwent surgical debridement and washout with removal of spinal metalwork to achieve source control. Intra-operative samples showed AFB on smears, and *M. abscessus* complex was identified on culture (Table 1) and whole genome sequencing (WGS); however, subspeciation could not be performed.

Her postoperative course was complicated by severe pancreatitis, requiring admission to the intensive care unit (ICU), which led to the discontinuation of tigecycline and bedaquiline, tapering of prednisolone, and the addition of cefoxitin. Due to the limited antibiotic options for *M. abscessus*, the isolate was sent to a research laboratory in Cleveland Veteran's Affairs (VA) Medical Center, Ohio, United States of America, for β -lactam synergy DST. To decrease net immunosuppression, prednisolone was tapered to 10 mg d^−1^. Following discharge from ICU, she developed new skin nodules on her extremities, a biopsy of which showed necrotizing granulomatous inflammation; however, mycobacterial culture of the biopsy was negative and blood cultures were negative. Repeat imaging revealed multiple paraspinal collections concerning for abscess (Fig. 2d). No other sources of infection were identified throughout the hospitalization. Once imipenem was available, cefoxitin was switched to dual β -lactam (DBL) therapy with imipenem plus ceftaroline (Fig. 1).

Two weeks after leaving the ICU, the patient suffered a cardiac arrest and died. Given the prolonged course of illness with multiple comorbidities, the patient's family decided not to proceed with a post-mortem examination.

## 3 Discussion

There has been an increase in the number of reports of NTM infections associated with “medical tourism” (i.e. travelling to another country to receive medical care), mainly for cosmetic and elective procedures (Cusumano et al., 2017). Infection can be acquired in these circumstances through inadequately sterilized equipment and/or the use of non-sterile water during surgical procedures, which was the likely mechanism of infection for our patient during her gastric sleeve surgery (Cusumano et al., 2017). This exposure coupled with her underlying immunosuppression led to her disseminated disease (To et al., 2020).

Although cases of vertebral osteomyelitis caused by *M. abscessus* have been described in the literature, it still remains an uncommon clinical presentation with limited data relating to the management of this infection (Rhodes et al., 2025; Omori et al., 2022). The lack of specific guidelines for the management of extra-pulmonary or disseminated *M. abscessus* leaves clinicians extrapolating from the American Thoracic Society (ATS)/European Respiratory Society (ERS)/European Society of Clinical Microbiology and the Infectious Diseases (ESCMID)/The Infectious Diseases Society of America (IDSA) guidelines for the treatment of NTM pulmonary disease (Benjamin et al., 2024; Griffith et al., 2007; Daley et al., 2020). The regimens endorsed by these guidelines favour the use of a macrolide, combined with amikacin, cefoxitin, or imipenem, with the goal of having at least three active drugs in the regime (Griffith et al., 2007). In our case, amikacin was the antimicrobial most consistently included given it is the most active of the parenteral agents (Griffith et al., 2007). Bedaquiline was included but later discontinued due to pancreatitis. A systematic review and meta-analysis by Omar et al. (2024) found that bedaquiline was effective in the treatment of extrapulmonary NTM infections (Omar et al., 2024). However, only six patients had extra-pulmonary NTM infection in their review, and they were all reported as “cured” when bedaquiline was included in their antimicrobial regimen. The true effectiveness of bedaquiline in the treatment of *M. abscessus* remains to be determined.

An important virulence factor of *M. abscessus* is its ability to form biofilms, which allows the persistence of infection (Lebeaux et al., 2014). In our case, the retained metalware from surgery to treat her acute cauda equina syndrome served as a reservoir for the relapsed infection after the patient's net immunosuppression was increased. The ATS and IDSA 2007 guidelines highlight that surgical management is an important aspect of treatment of extrapulmonary NTM infection and that removal of foreign material is the key to microbiological eradication (Griffith et al., 2007).

Three subspecies of *M. abscessus* have been identified: *M. abscessus* subsp. *abscessus*, *M. abscessus* subsp. *massiliense*, and *M. abscessus* subsp. *bolletii* (Lee et al., 2015). In subsp. *abscessus* and *bolletii*, the presence of an erm (41) gene gives rise to inducible macrolide resistance. However, in subsp. *massiliense*, the erm (41) gene is non-functional (Lee et al., 2015); hence, infections caused by this subspecies have better outcomes (Choi et al., 2012). For our patient, subspeciation at the external reference laboratory could not be performed.

Another challenge in the treatment of *M. abscessus* infection is the lack of correlation between in vitro DST and clinical outcomes in vivo. For our patient, the drug-induced pancreatitis left limited treatment options for the *M. abscessus* infection, so we explored the use of DBL therapy given established high synergistic in vitro activity. Though drug susceptibility testing for DBL is not clinically validated, there have been favourable reports of clinical success of dual β -lactam therapy for *M. abscessus* as part of a multidrug regime (Alahmdi et al., 2023). The myriad of *M. abscessus* clinical syndromes may respond differently to the same treatments due to an unknown varied correlation between in vitro DST and clinical outcomes in vivo (Griffith et al., 2007).

## 4 Conclusions

With the rising prevalence of *M. abscessus* infection, more data are needed to determine the optimal antimicrobial treatment regime for extrapulmonary or disseminated disease given the current lack of guidelines. Our case highlights several essential interventions that may increase the likelihood of successful treatment of disseminated NTM disease, namely the need to decrease net immunosuppression, how removal of retained metalwork due to biofilm formation is paramount to achieve adequate source control and prevent relapses, and finally the challenges of maintaining a robust antimicrobial regime throughout prolonged courses of therapy where side effects commonly develop.

## Data Availability

No data sets were used in this article.
